# A resilience view on health system resilience: a scoping review of empirical studies and reviews

**DOI:** 10.1186/s12913-023-10022-8

**Published:** 2023-11-24

**Authors:** Samantha Copeland, Saba Hinrichs-Krapels, Federica Fecondo, Esteban Ralon Santizo, Roland Bal, Tina Comes

**Affiliations:** 1 Faculty of Technology, Policy and Management, Delft University of Technology, Jaffalaan 5, 2628 BX Delft, The Netherlands; 2 Erasmus School of Health Policy & Management, Erasmus University Rotterdam, Burg. Oudlaan 50, Rotterdam, The Netherlands

**Keywords:** Resilience, Health systems, Resilience engineering, Socio-ecological resilience, Community resilience

## Abstract

**Background:**

Prompted by recent shocks and stresses to health systems globally, various studies have emerged on health system resilience. Our aim is to describe how health system resilience is operationalised within empirical studies and previous reviews. We compare these to the core conceptualisations and characteristics of resilience in a broader set of domains (specifically, engineering, socio-ecological, organisational and community resilience concepts), and trace the different schools, concepts and applications of resilience across the health literature.

**Methods:**

We searched the Pubmed database for concepts related to ‘resilience’ and ‘health systems’. Two separate analyses were conducted for included studies: a total of *n* = 87 empirical studies on health system resilience were characterised according to part of health systems covered, type of threat, resilience phase, resilience paradigm, and approaches to building resilience; and a total of *n* = 30 reviews received full-text review and characterised according to type of review, resilience concepts identified in the review, and theoretical framework or underlying resilience conceptualisation.

**Results:**

The intersection of health and resilience clearly has gained importance in the academic discourse with most papers published since 2018 in a variety of journals and in response to external threats, or in reference to more frequent hospital crisis management. Most studies focus on either resilience of health systems generally (and thereby responding to an external shock or stress), or on resilience within hospitals (and thereby to regular shocks and operations). Less attention has been given to community-based and primary care, whether formal or informal. While most publications do not make the research paradigm explicit, ‘resilience engineering’ is the most prominent one, followed by ‘community resilience’ and ‘organisational resilience’. The social-ecological systems roots of resilience find the least application, confirming our findings of the limited application of the concept of transformation in the health resilience literature.

**Conclusions:**

Our review shows that the field is fragmented, especially in the use of resilience paradigms and approaches from non-health resilience domains, and the health system settings in which these are used. This fragmentation and siloed approach can be problematic given the connections within and between the complex and adaptive health systems, ranging from community actors to local, regional, or national public health organisations to secondary care. Without a comprehensive definition and framework that captures these interdependencies, operationalising, measuring and improving resilience remains challenging.

**Supplementary Information:**

The online version contains supplementary material available at 10.1186/s12913-023-10022-8.

## Introduction

### Background and context to this review

The past 10 years have challenged health systems globally with several public health emergencies (COVID-19, SARS, Ebola Virus Disease), environmental disasters (flooding, hurricanes and earthquakes), as well as war and conflict shocks. Although health providers often deal with crises, these large-scale emergencies have revealed a need to deal with unprecedented care demand and shortages of staff, supplies and infrastructure. Beyond the direct effects and disruptions of each crisis, there are cascading effects such as delays in routine and non-emergency care. When these crises start to spread across political borders, there are governance, financial and cultural barriers that affect the ability to provide care equitably to all those in need.

These sudden shocks and disruptions have prompted policymakers, practitioners, and scholars to learn how to increase the resilience of health systems. In recent research, perhaps prompted by COVID-19, there is has been a surge of interest in the concept of resilience, and many reviews have already been conducted on this topic, exploring the presence of resilience in empirical studies, or whether we can learn from the concepts of resilience generally to improve health system performance. For example, Biddle et al. 2020 provide a narrative review of resilience concepts in empirical studies, but provide little in-depth characterisation of resilience characteristics and their relevant capacities, or their applications across different health systems, which could provide useful learning on how to operationalise resilience in different health system contexts [1]. As Forsgren et al. (2022, this issue) suggest, this focus on the theoretical has led to a lack of knowledge about which strategies for building resilience have been successful, a gap they seek to close [2]. Similarly, Khalil et al. (2022) point out the need to operationalise resilience concepts into healthcare practice [3]. However, what is missing is a thorough analysis of what resilience paradigms and concepts outside of the health domain can offer health systems research and practice. Wiig and O’Hara offer a particularly useful starting point with their analysis of the impact that resilience engineering concepts are having on health systems research [4]. Our aim here is to augment existing reviews on resilience in health systems, and to specifically compare the way resilience is operationalised in empirical studies to the core paradigms and conceptualisations of resilience from a broader set of domains (specifically, engineering sciences, socio-ecological sciences, organisational resilience, and community resilience). We thus take up the unique task, to review the paradigms and approaches to resilience, and map them on the different subsystems of the health system to which they are applied. Rather than bypass the possible variations in conceptual frameworks, we sought to track them; and thereby identify the relationship between conceptual origins showed in the literature, which parts of the health system they focus on, and the aspects of resilience used.

To achieve this objective, we had two specific sub-objectives which guided our search and review method:

1. To characterise the empirical studies dealing with resilience in health systems by (i) types of disasters, threats or events that have triggered these studies, (ii) the part of the health system covered, and (iii) the resilience concepts used in these studies.
2. To provide a review of reviews to show the various resilience paradigms and conceptualisations used across the health literature already synthesised by existing reviews.

### Conceptualisations of resilience in other domains influencing our review

The word resilience stems from the Latin resilio, or ‘to jump back’ [5]. Resilience has double roots in socio-ecological systems and in psychology. In psychology, research has focused on the ability of individuals to deal with trauma and extremely adverse events (Comes et al., 2019 [6]). Studies focus on personality traits that help defend against exposure to extreme stress; this includes aspects such as meaningful purpose, agency and a growth mindset [7]. Taking a systems- rather than an individual perspective, in this paper we do not focus on psychological resilience.

In socio-ecological systems, the concept of resilience was introduced in Holling’s seminal paper to characterise the ability of a system to evolve and adapt under shocks and stresses [8]. Holling’s work inspired a rich body of work in fields ranging from resilience of ecosystems to climate adaptation [9, 10]. Resilience in this realm is often defined as “*“the capacity of a system to absorb disturbance and reorganize while undergoing change so as to still retain essentially the same function, structure, identity, and feedbacks*” [11], or the ability to persist by changing. This evolutionary perspective stresses the emergence of systems properties, and the rise of rare events, which “*can unpredictably shape structure at critical times or at locations of increased vulnerability*” [12]. This notion of resilience, however, is in contrast with the need for planning and decision-making, and does not fit the long lead-times and life cycles that are typical for decisions in health care planning and management.

In engineering, resilience is largely used to refer to the ability of some system to rapidly return to a single state of equilibrium or stability after a disruption, thereby ensuring continued functioning and rapid recovery from stress and disturbances [13, 14]. Often, engineering resilience is associated with robustness, broadly understood as the ability to withstand shocks and ‘bounce back’ to the same performance level. These principles have been instrumental in identifying critical points in complex, interdependent infrastructure networks [15], analysing disruptions [16] or for designing new infrastructures and tools [17]. However, these approaches do not explicitly incorporate aspects of adaptation and transformation that socio-ecological systems or community resilience encompasses.

From the perspective of social-ecological resilience, which puts forward the concept of resilience as a non-equilibrium notion, there are important links to participation and self-organisation that are common to community resilience and the social capital theories of resilience [18, 19]. These concepts focus on the networked sets of capacities that a community or organisation can generate, in order to be(come) resilient [20]. While community resilience focuses on the adaptive capacity of communities and their abilities to respond [20], organisational resilience seeks to explain the features of highly resilient organisations [21, 22]. These concepts are also prominent in the disaster resilience literature, where resilience has been promoted as a way to analyse and understand the reaction of a system to a hazardous event, promoting activities such as improving coping capacities and livelihoods [23].

Overall, we observe three major principles that are meant to improve resilience: reducing impacts or consequences (robustness), reducing recovery time (absorption), and reducing future vulnerabilities (adaptation and transformation). These principles are represented to a different degree in the four paradigms that we are investigating here: socio-ecological systems resilience [8], engineering resilience [14, 24], community resilience [20], and organisational resilience [21, 22].

For our study, we sought to look for instances in which these concepts were used, addressed, or referred to, either in the empirical studies (aim 1 above) or in the reviews (aim 2 above).

### Methods

**Fig. 1:**
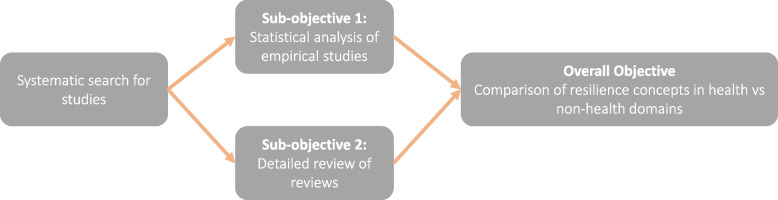
Overview of the two sub-objectives for our study

We describe our work as a scoping review of empirical studies and reviews, which we explain as follows: We first searched for any studies relating to resilience and its accompanying definitions in the health literature, and applied inclusion/exclusion criteria (see full details below). We then divided our included studies into two categories to match our sub-objectives above: empirical or non-review studies were analysed according to the characteristics described in sub-objective (1) above, while all reviews were reviewed separately and key resilience themes and concepts were extracted for sub-objective (2). The results from both components were analysed together to satisfy our overarching objective. These components are conceptualised in Fig. 1.

### Search method

We expanded the search string and method used by Turenne et al. 2019 [25], who provided a conceptual analysis of health systems resilience, by adding broader search terms that enabled us to include regional, local or care provider-based empirical studies. We also limited our search to PubMed, as our focus was to identify specifically how the health literature conceptualises and uses ‘resilience’ and compare this analysis to other disciplinary domains in our analysis and discussion. We conducted our search on 16 June 2021 which yielded 2773 results (see Supplementary file A for PRISMA flowchart); and the full search string is provided below:

- (((“resilien*”[Title/Abstract]) OR ("coping strateg*"[Title/Abstract]) OR ("system responsiveness"[Title/Abstract]) OR ("system adaptation"[Title/Abstract])).
- AND ( ("health* system*" [Title/Abstract]) OR ( (“health systems plans" [MeSH Terms]) OR ( "Health Planning/organization and administration"[Mesh])) OR ("Public Health/organization and administration"[Mesh]) OR ( "Organization and Administration/organization and administration"[Mesh] OR "Organization and Administration/prevention and control"[Mesh] OR "Organization and Administration/supply and distribution"[Mesh]) OR ("comprehensive health care/organization and administration"[Mesh]) OR ("Public Health Administration"[MeSH]) OR ("Public Health Systems Research"[MeSH]) OR ("health policy"[MeSH]) OR (national health programs/organization and administration[MeSH]) OR ("efficiency, organizational" [MeSH]) OR ("Health Services/organization and administration"[MeSH]))).
- AND ("english" [Language]).

To remain up to date with the latest literature, and in response to helpful feedback from one of our reviewers, we updated our search in June 2023 which yielded a further 796 results. These are not included in the empirical study review (sub-objective (1) in Fig. 1), but we did include the extra *n* = 23 reviews we found in the full-text review analysis (sub-objective (2)).

### Eligibility criteria

For the analysis of empirical studies (sub-objective (1) in Fig. 1), we included studies if they met the following criteria:

- **Context:** The study is conducted within any part of the health system (including primary care or social care settings, national decision-making, public health local or regional authorities). Individual psychological resilience (of patients or of the workforce) were excluded, unless the study explicitly related such individual resilience as a contributor to the resilience of the system.
- **Process:** The study relates to any aspect of resilience including, for example, adaptation, coping mechanisms, learning from a shock or disaster (see below for ‘resilience concepts’ used in data extraction and analysis).
- **Study type:** Commentaries, editorials, news articles and conference proceedings were excluded.
- **Language:** Only studies in English were included.
- **Time period:** No date limitations were set.

For eligibility in the full-text review of reviews (sub-objective 2 above), we applied the same criteria, except for study type, since this had to be a review with an included search and analysis method (e.g., scoping review, systematic review, narrative review).

### Study selection

We used the platform ‘Rayyan’ for screening the titles and abstracts. Three reviewers piloted the screening based on the inclusion/exclusion eligibility criteria. Two reviewers rescreened these relevant/possibly relevant records and we resolved the disagreements in group meetings. We followed arbitration by a third reviewer.

Following study selection, the studies were divided into two categories (Fig. 1): (a) empirical studies that concerned resilience of the health system, and (b) reviews (any method included). This resulted in *n* = 87 articles included in part (a) and *n* = 30 articles included in part (b) the review of reviews (see Supplementary file A for our adapted PRISMA-Scoping reviews flowchart). The following steps (data extraction and analysis) were conducted separately and their associated methods are reported in two parts below.

### Data extraction and analysis

- Data analysis for sub-objective 1: statistical analysis of empirical studies.

While the studies included in these statistical analyses were not reviewed in full, the following data from these articles were extracted:

- **General study publication information:** This includes publication date, journal, authors, title, location of study (country).
- **Types of threat:** This refers to the type of threat, event or disaster studied, including COVID-19, Ebola, environmental disasters, etc.
- **Part of the health system covered in study:** This included health system (general/unspecified), community health workforce, primary care, community formal or informal actors (non-health), secondary care (hospital), public health (national, prevention), public health (national), regional/local public health organisations.
- **Resilience paradigms:** To understand the use and evolution of the resilience concept and its characteristics within health systems research, we analysed the articles according to the underlying resilience research paradigm. We searched for the different resilience domain perspectives as outlined above. As we were aiming to embed the health resilience studies into the broader resilience discourse, we here focused on links to the existing fields, rather than on the emerging literature on health resilience. The categories included socio-ecological systems resilience, engineering resilience, community resilience, and organisational resilience.
- **Resilience aspects:** We analysed the resilience phase or aspect considered. Following Manyena et al. (2019) [23] and their comprehensive review of the resilience literature, we distinguish preventive, preparedness, absorptive, adaptive and transformative capacities. We use, whenever possible, the standards defined by UNISDR terminology^1^ and combine these with recent a recent policy documents for the EU [26] and the IPCC Glossary^2^:
  - Prevention: “activities to provide outright avoidance of the adverse impact of hazards” (UNISDR)
  - Preparedness: “activities and measures taken in advance to ensure effective response to the impact of a hazard” (UNISDR)
  - Absorption: “ability of a system to keep or rapidly recover the same level of performance and service delivery (in terms of quantity, quality, and equity)” [26]
  - Adaptation: “process of adjustment to changing conditions, including risks and crises” [26]
  - Transformation: “a profound and often deliberate shift initiated by communities toward sustainability, facilitated by changes in individual and collective values and behaviours” (IPCC).
- **Resilience approach:** Subsequently, we also analysed the approaches that were put forward or studied as a means to achieve resilience (e.g., robustness, agility, redundancy). Rather than diving deeply into the use and meaning of each concept, we here compare how the concepts are used across the different literatures, and which terms dominate.

Further, we followed Meerow et al.’s [27] approach in analysing resilience *of what* and *to what.* In the ‘*of what’* category, we analysed which parts of the health system were studied, ranging from specific departments within a hospital, to the public health system. Under ‘*to what’* we analysed the shocks or stresses that the system under consideration was supposed to, and the drivers for resilience. To understand how the different concepts are applied across paradigms and domains, we developed heat maps that show the frequency of co-occurrence of different terms.

We designed and tested the data extraction form in a spreadsheet shared via Google Sheets to enter: author-title of the review, year and location(s), country in which the empirical study was conducted, threat (*to what?*), the part of the health system that was studied (*of what?*), the underlying theoretical paradigm used, the resilience phase, and the concepts that were referred to as means to improve resilience. We also indicated if the related choices were not made specific or could not be inferred from the manuscript. To capture intersections between the concepts, and understand how different capacities are co-evolving or co-used, we analysed and counted *all* concepts that a paper touched upon, i.e., if a paper referred to e.g., absorption and adaptation, we counted it under both categories.

Where possible, we inferred paradigms and concepts as mentioned from the abstract. If that was not possible, the full papers were scrutinised for additional information. If the category could not be detected, we labelled the paper as ‘not specified’. In addition, because of the breadth of the field, we grouped papers where possible. For instance, papers that referred to ‘rapidly bouncing back’ were categorised under the resilience engineering paradigm.

(b) Data extraction for sub-objective 2: review of reviews.

The reviews included in our study (*n* = 30) were reviewed via full-text review. We designed and tested a separate extraction form in a spreadsheet shared via Google sheets to include: type of review, resilience concepts identified in the review, and theoretical framework or underlying resilience framework or definition.

## Results

We present our results in two sections. In part (a) we describe the nature of the included empirical studies that address resilience in health systems. This includes the types of disasters or events that are either used for data or context for these studies, their chronology, the part of health system covered by the studies, methods used, and resulting knowledge contribution of the study. In part (b) we present the results of our full scoping review of reviews, focussing in particular on the way resilience is conceptualised in these papers. In the discussion following this section, we compare and contrast these conceptualisations to the principles of resilience identified within part (1).

### (a) Describing empirical studies addressing resilience within health systems

We included a total of *n* = 87 articles in the descriptive analysis, available in full in a table in Supplementary File C.

#### Nature of empirical studies on resilience in health systems

**Fig. 2:**
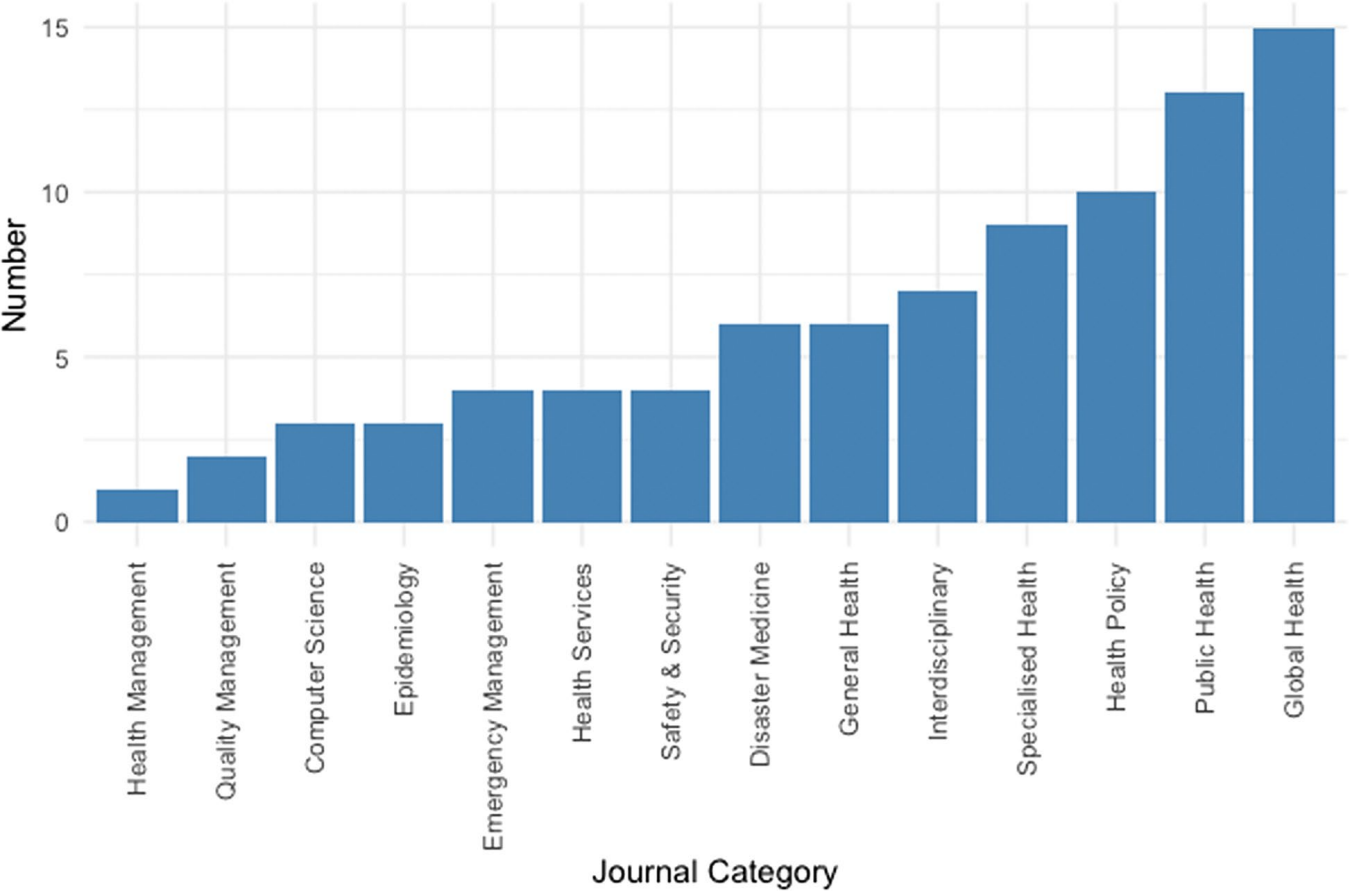
Distribution of journal categories considered in this review (*n *= 87)

Our included articles are drawn from a broad spectrum of journals covering different fields and domains within healthcare (Fig. 2), with the highest number of publications in global health (*n* = 15, 17%) and public health (*n* = 13, 15%). These studies are also published in interdisciplinary journals, or in other domains, such as emergency management (*n* = 4, 5%) or computer science (*n* = 3, 3%).

#### Location and setting of empirical studies on resilience in health systems

**Fig. 3:**
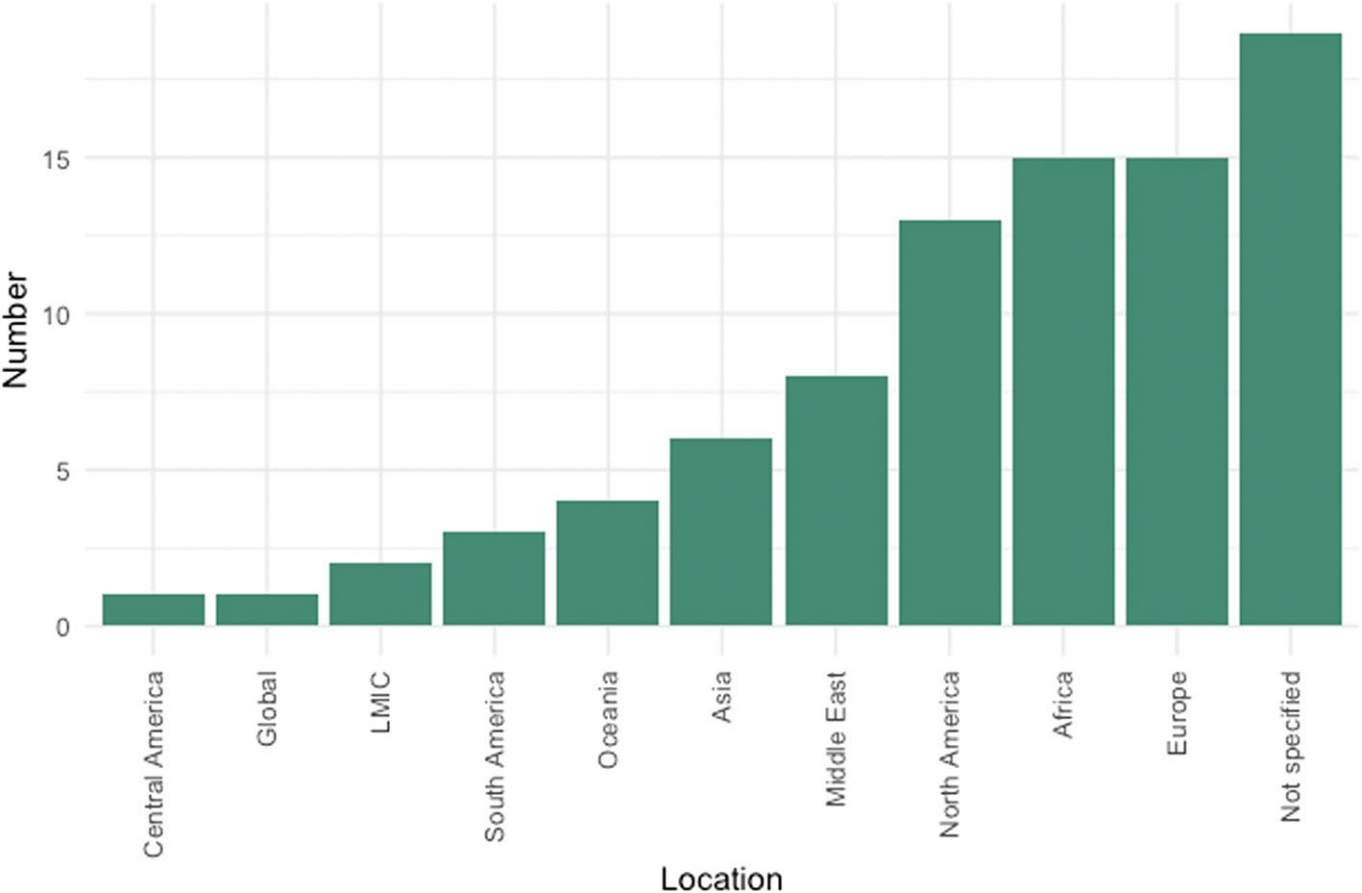
Distribution of study locations (*n* = 87)

The settings in which these included studies were conducted were distributed fairly evenly across Europe (*n* = 15, 17%), Africa (*n* = 15, 17%), and North America (*n* = 13, 15%), see Fig. 3. For Asia, we distinguished the Middle East (*n* = 8, 9%), where papers often focus on conflict and refugees (e.g. [28, 29], from South Eastern Asia and China (*n* = 6, 7%), where studies largely focused on emergency management departments [30, 31]. Fewer studies were conducted in Oceania (*n* = 4, 5%) South America (*n* = 3, 3%) and Central America (*n* = 1, 1%). Few papers reported to focus generally on low- and middle-income countries (LMICs) (*n* = 2, 2%) or global health systems issues (*n* = 1, 1%). A total of 18 papers (21%) did not specify the location of study within the title or abstract.

#### Threats prompting empirical studies on resilience

**Fig. 4:**
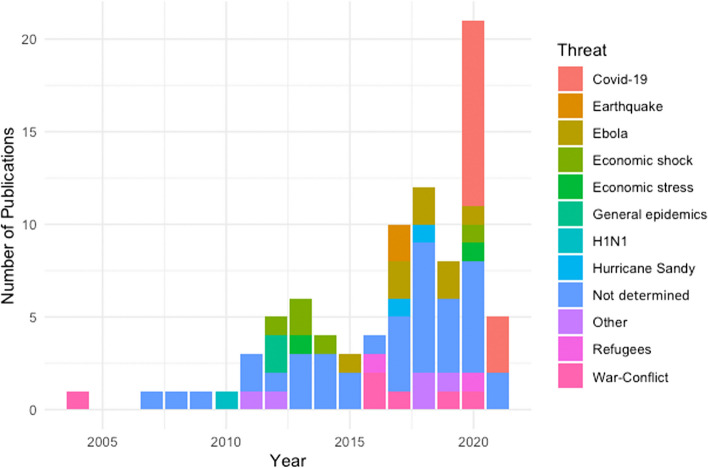
Identified literature on health system resilience (*n* = 87) organised by threat or type of challenge and year (from 2004 until June 2021)

By categorising the studies according to their year of publication and the associated ‘threat’ (disaster, emergency etc.) that was studied, we were able to observe patterns in how these threats prompted resilience research over time (Fig. 4). The intersection of health and resilience clearly has gained importance in the academic discourse with the majority of papers published since 2018 (*n* = 47, 54%). Most recently, the COVID-19 pandemic created another peak in health systems research (starting 2020, *n* = 21 or 24%), after a slight drop in 2019. The 2021 data covers the papers published up to June 2021, when we conducted our search for empirical studies.

A majority of publications (*n* = 49, 56%) made specific the resilience challenge addressed, while 38 publications were generally referring to health systems resilience, driven by the general complexity and uncertainty that the health system is exposed to, but without referring to a clear threat or challenge. For the publications that refer to a threat, infectious diseases and epidemics (*n* = 24, 27%) formed the largest group of publications. Clearly, this category was dominated by COVID-19 (*n* = 13) and Ebola (*n* = 8) with other infectious diseases playing a minor role.

Many of the COVID-19 publications reported on the lessons learnt from the first wave of the pandemic across different countries [32–35]. The publications about Ebola (which gained prominence in 2015), were published from 2015 to 2020, speaking to the long-term challenges to the health system. While the initial publications focus on the immediate impact [36], the later studies shift to the development of the health system [37, 38] and community resilience or community health workers [39–41].

Somewhat surprisingly, natural disasters find relatively few mentions with a total of 5 publications (6%) that cover Hurricane Sandy e.g., [42, 43] and earthquakes in Central America (e.g., Haiti [44]) and South Eastern Asia (Fukushima e.g., [45]). Economic stresses and shocks (*n* = 7, 8%) have gained importance in the aftermath of the 2011 financial crisis across geographical locations (e.g., [46–49]). The ongoing war and conflicts in the Middle East and the subsequent refugee crises have inspired a range of health resilience papers since 2016 (*n* = 9, 10%) [28, 29, 50].

#### Components of the health system covered by empirical studies, compared to threats

**Fig. 5:**
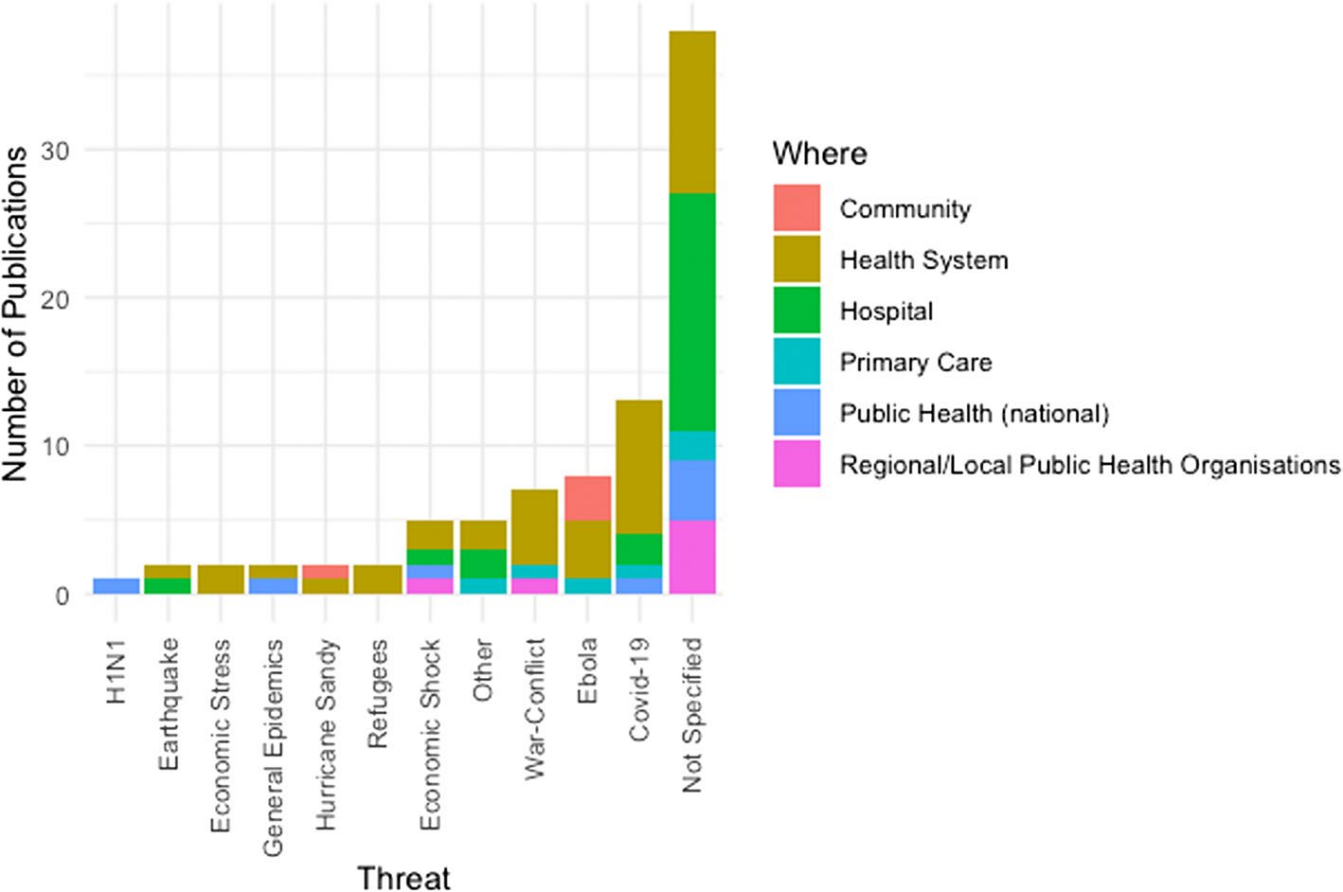
Literature on health resilience (*n* = 87) organised by location in the health system (resilience of what?) and threat or type of challenge (resilience to what?)

Focusing on the question which parts of the health systems are the objects of investigation, Fig. 5 shows that most studies focus on the health system generally, without further specification (*n* = 40, 46%), followed by secondary care (specifically, hospitals; *n* = 21, 24%). The articles focussed on health systems generally cover diverse challenges and specific threats ranging from conflict and economic crises to antimicrobial resistance [51–54]. In contrast, the resilience of hospitals is often studied in general terms, with the majority of papers studying the regular disruptions, uncertainties and complexities with which a hospital is confronted [55–58], rather than specific and/or external shocks or stresses. Primary care (*n* = 9, 10%) is mostly discussed in a post-disaster or conflict context [53, 59, 60]. National public health organisations (*n* = 8, 9%) [61], regional and local public health care organisations (*n* = 6, 7%) [62] and community actors (formal, informal and workforce) (*n* = 4, 5%) (eg [40]) have received less attention. Figure 5 shows that the publications on communities were driven by the literature on Ebola (e.g. [39, 40, 63]), which also have promoted a shift towards community.

#### Aspects and phases of resilience used in empirical studies

Conventionally, the health resilience literature distinguishes three main capacities or outcomes: absorption, adaptation and transformation [1, 64]. However, our findings show that many of the empirical studies we found focussing on resilience—given that they are rooted in the risk, safety, and emergency management domain—also focus on prevention and control (e.g. [31, 46]) as well as preparedness [65–67] as key capacities and outcomes. Especially in response to COVID-19, several authors focus on preparing for or preventing pandemics (e.g., [33, 68, 69]). While conventionally, these outcomes are considered as a part of risk management rather than resilience [26], the health resilience literature integrates both realms under the umbrella of resilience.

**Fig. 6:**
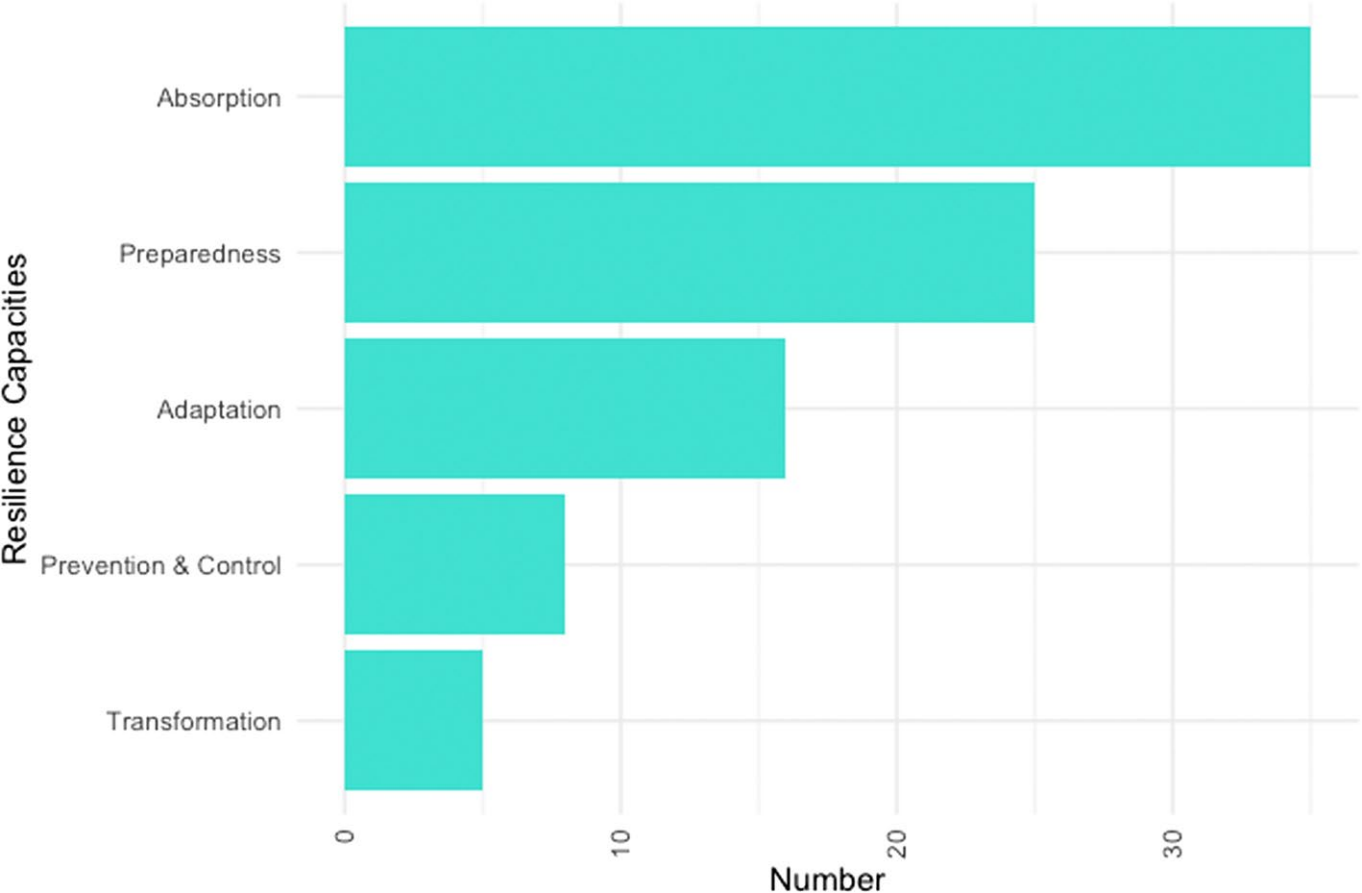
Analysis of specific resilience capacities or outcomes (*n* = 87)

Figure 6 shows that the idea of absorption, or rapidly bouncing back, is the most used resilience aspect (*n* = 35, 40%) with a wide range of applications, ranging from communities recovering from natural disasters [42] to the ability of emergency departments to absorb a surge of patients [30, 70] or external disturbances [71]. Preparedness (*n* = 25, 29%) and adaptation (*n* = 15, 17%) follow. Prevention & control (*n* = 8, 9%) are primarily referred to in literature that describes resilience to epidemics and infectious disease (see above). Transformation (*n* = 5, 6%) has received limited attention [49], often in conjunction with other qualities, such as adaptation (e.g., [59]. And while the resilience literature often stresses the need to absorb, adapt *and* recover, only two publications (2%) refer to all three capacities [28, 50]. Strikingly, both of these publications focus on the situation of Syrian refugees.

#### Underlying resilience paradigm or disciplinary contributions in empirical studies

**Fig. 7:**
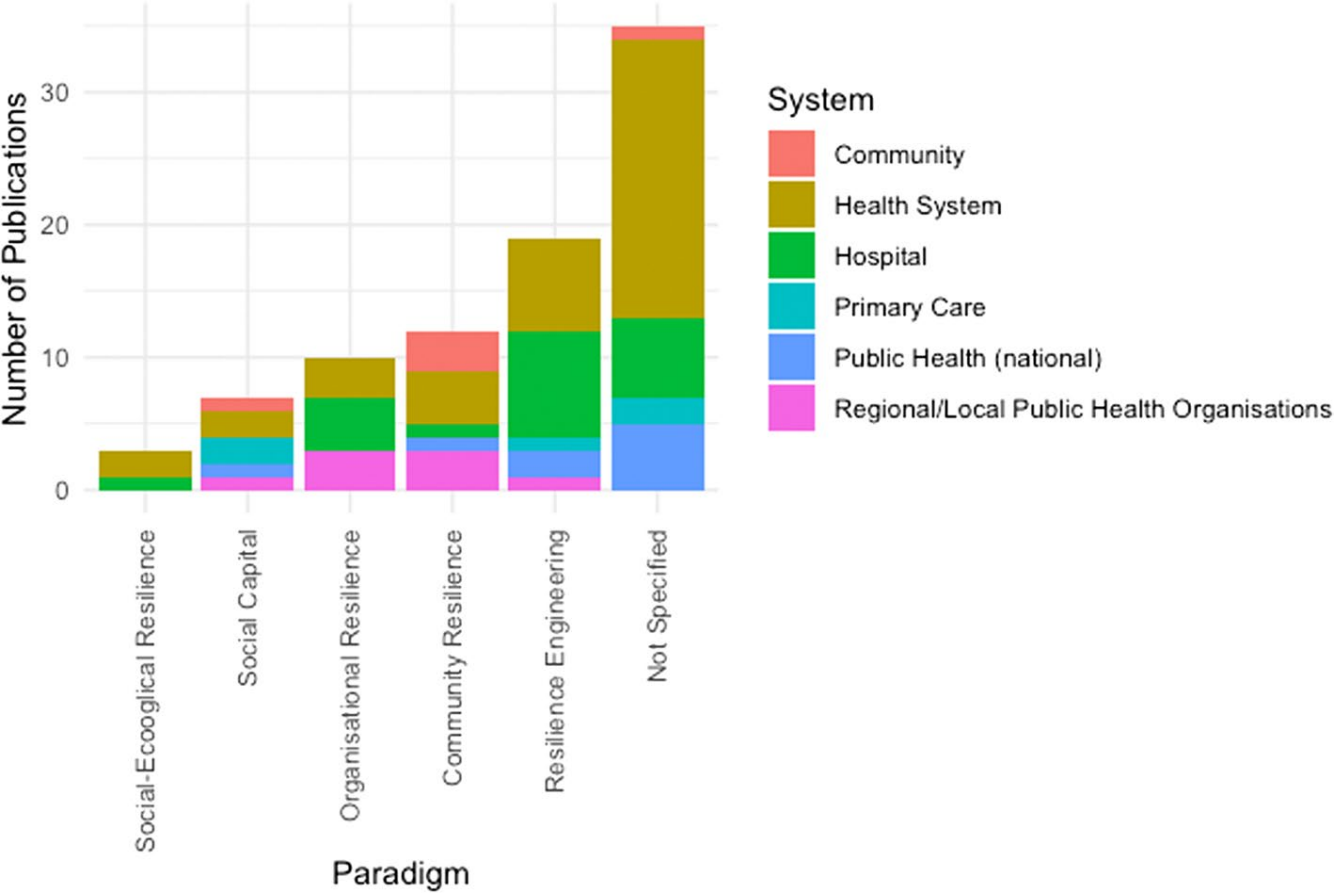
Literature on health resilience organised by location in the health subsystem (resilience of what?) and resilience paradigm (*n* = 87)

To track how these different research traditions and paradigms are influencing the field of health resilience, we analysed the publications for mentions of the underlying field. Most publications do not make the research tradition in which they are embedded specific (*n* = 36, 41%, e.g. [72–74]). Of those that make explicit an underlying research paradigm, resilience engineering is the most prominent (*n* = 19, 22%, e.g. [60, 75, 76]). This finding is also in line with the prominence of ‘absorption’ or the rapid response to shocks discussed earlier. Figure 7 highlights that resilience engineering is applied throughout most health subsystems, but is especially prominent in secondary care (hospitals), e.g. [30, 56, 77]. Community resilience and organisational resilience are following (*n* = 12 and *n* = 10 respectively). Community resilience approaches find application in both local and regional public health organisations [65, 78], in analyses of the formal and informal community actors or workforce [41, 63] as well as in the study of the health system as a whole [37, 79]. Organisational resilience is applied to the different levels of public health organisations (local / regional) [31, 80] as well as secondary care (hospitals) [66, 70, 81]. The social-ecological systems roots of resilience find the least application, both in the health system [51] as well as within hospitals [47], confirming our findings of the limited application of transformation in the health resilience literature.

#### Approach to developing and building resilience

Even broader than the different categories and capacities that constitute resilience are the approaches that are used or analysed to improve or manage resilience in health systems. Most prominently described is the need for surge capacity to respond to a rapid shock such as a natural disaster or an epidemic (*n* = 18, 21%) (e.g., [45, 79, 82], followed by resilience capacity, as a generic umbrella term (*n* = 12, 14%, e.g. [28, 37]), trust (*n* = 8, 9% [83]) and leadership (*n* = 7, 8% [84]). Other concepts that are broadly used in resilience management or resilience engineering receive surprisingly little attention, such as robustness [85], redundancy [71], flexibility [57] or agility [34] (all below five mentions).

**Fig. 8:**
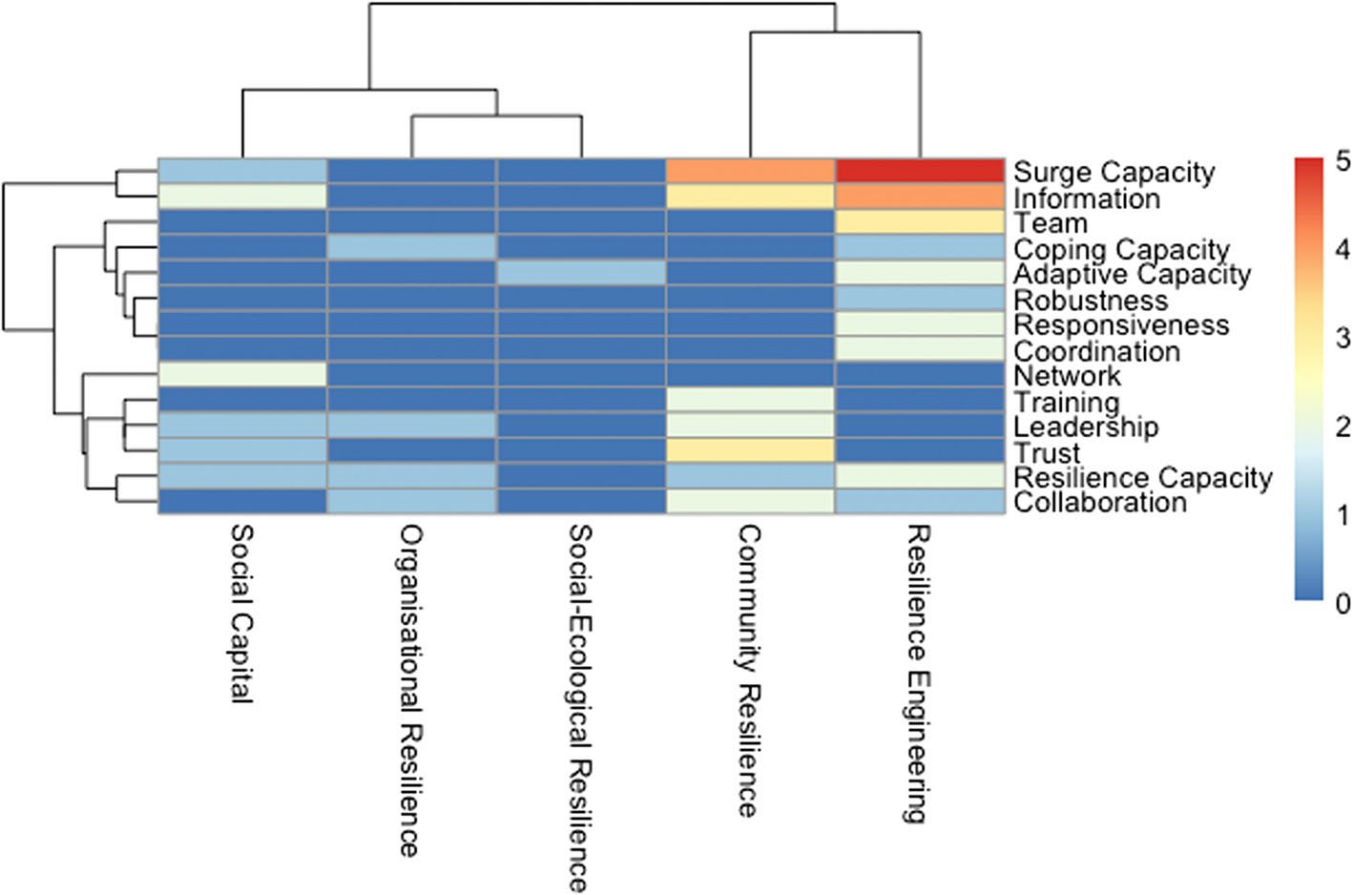
Heatmap and dendogram of the approaches to build and manage resilience for the different resilience paradigms. (socio-ecological systems—social-ecological resilience; ORG—organisational resilience; SoCap—Social Capital; COMM—community resilience; RE—resilience engineering). Mapping only for papers that mention both resilience paradigm and approach

Figure 8 shows the clear divide of the studied approaches to resilience by the different resilience paradigms, also illustrated by the dendrogram, showing the hierarchical relationships between the different uses of the concepts in the discourse. While resilience engineering emphasises the need for concepts and terms such as ‘coordination’, ‘robustness’, ‘flexibility’, ‘teams’ and different ‘capacities’, the discourse in the literature that is rooted in community resilience stresses ‘collaboration’, ‘trust’, ‘training’, and ‘leadership’. Both resilience engineering and community resilience-oriented approaches acknowledge the need for ‘information’ and ‘surge capacity’. Social capital-oriented literature is focusing primarily on the role of ‘networks’, while the organisational and social-ecological systems resilience literatures stress the need for ‘diversity’.

### (b) Describing how resilience is conceptualised in the health domain based on reviews

We included a total of *n* = 30 articles in the review of reviews, included in Table 1 (*n* = 17 up to June 2021, matching the date of the empirical studies analysed in (a), plus a further *n* = 23 reviews published after June 2021 up to June 2023). These included systematic reviews (*n* = 11), scoping reviews and other or non-systematic. Not surprisingly, most of the reviews were published more recently: 2022 (*n* = 10), 2021 (*n* = 5), 2020 (*n* = 4). Most of them identify what constitutes ‘resilience’ in health systems, but only few refer to conceptualisations in non-health domains such as engineering and other sciences for example, Biddle et al. 2020 [1] and Hess et al. (2012) [86]. Many of the earlier reviews refer to the definition of resilience by Kruk et al. (2017), which includes being adaptive, self-regulating, diverse, aware, and integrated [87]. These characteristics draw on socio-ecological systems paradigms but this allusion is not explicitly mentioned in the reviews using them as a conceptual framework. In later reviews, authors mostly appealed to the domains of resilience identified by Blanchet et al. (2017) [88], which includes the concept of transformation, but that aspect is not in fact widely used (e.g. [1]).

**Table 1:** Table of reviews used for full-text review (*n* = 30)

**Included review reference**	**Type of review**	**Resilience phases, concepts and paradigms indicated in the paper (and underlying framework, if specified)**
Bayntun, 2012 [89]	systematic review	(includes reference to general WHO framework)
Hess et al. 2012 [86]	literature review	adaptive capacity
Bayntun et al. 2012 [90]	systematic review	disaster management as health system strengthening; (includes reference to WHO framework/checklist)
Zhong et al. 2014 [91]	literature review	preparedness, adaptation, absorption
Curtis et al. 2017 [92]	structured review	preparedness, adaptation (eg building design); role of community
McDarby et al. 2019 [93]	scoping review	(includes reference to Kruk et al. 2017 [87])
Turenne et al. 2019 [25]	scoping review	adaptation, maintenance, absorption, learning, transformation, withstanding and responding to shocks
Khan et al. 2019 [67]	review, followed by delphi	preparedness, prevention/control; community; socio-ecological systems; organisational
Nuzzo et al. 2019 [94]	scoping review	transformation, preparedness; (includes reference to Kruk et al. 2017 [87])
Chamberland-Rowe et al. 2019 [95]	systematic review	preparedness, adaptation; transformation (opportunism), absorption; (includes reference to Kruk et al. 2017 [87])
Ayanore et al. 2019 [96]	systematic review	not specific [related to preparedness]; (includes reference to Kruk et al. 2017 [87])
Haldane et al. 2020 [97]	rapid review and narrative synthesis	surge capacity/absorption; control; organisational resilience
Fridell et al. 2020 [98]	scoping review	adaptation, maintenance, absorption, learning, transformation, withstanding and responding to shocks; resilience engineering
Biddle et al. 2020 [1]	systematic review	absorption, adaptation, transformation; (includes reference to Blanchet et al. 2017 [88])
Meyer et al. 2020 [99]	scoping review	preparedness; (includes reference to Kruk et al. 2017 [87])
Haldane et al. 2021 [79]	literature review	adaptation (toward equity - almost transformation), (not absorption), adapted WHO framework; community engagement; socio-ecological systems; social capital
Grimm et al. 2021 [100]	evidence synthesis	adaptation; (includes reference to Kruk et al. 2017 [87])
Luke et al. 2021 [101]	scoping review	(hospital) socio-natural disaster resilience; structural & non-structural resilience, infrastructure resilience; (includes reference to WHO hospital safety index tool and 'Senai' framework for disaster risk reduction)
Alehi et al. 2021 [102]	systematic review	workforce resilience (organisational)
Hasan et al. 2021 [103]	scoping review	responsiveness
Sutherns and Olivier. 2022 [104]	scoping review	responsiveness mechanisms; response [to public voice via policy]; (includes references to WHO responsivenes)
Foroughi et al. 2022 [105]	systematic review + critical interpretive synthesis	absorptive, adaptive, transformative; (includes references to Barasa et al. 2018 [106]; Thomas et al. [114])
Foroughi et al. 2022 [107]	systematic review	adapative capacity, transformation, absorptive
Forsgren et al. 2022 [2]	scoping review	workforce resilience, governance, preparedness, everyday resilience, strengthening, learning, [adaptive capacity in relation to chronic stresses], leadership; (includes reference to Barasa et al. 2018 [106])
Khalil et al. 2022 [3]	scoping review + interviews	strengthening resilience; hospital resilience; everyday resilience, functionality and safety; in the literature found: absorptive, adaptive, transformative + learning as key aspects; (prevention + preparedness)
Falope et al. 2022 [108]	systematic review	elements of resiliency (being aware, diverse, self-regulating, integrated, and adaptive); (includes reference to Kruk et al. 2017 [87])
Thu et al. 2022 [109]	literature review	absorbtion, adaptation, transformation; (includes reference to Blanchet et al. 2017 [88])
Fleming et al. 2022 [110]	systematic review	absorption, adaptation, transformation + dynamic shock cycle; (includes reference to Thomas et al. 2020 [114])
McDarby et al. 2022 [111]	rapid literature review	(embedding resilience into) strengthening, learning, measurable/tangible (lit reviews finds common: awareness, mobilization, self-regulation, integration, diversity, transformation)
Ismail et al. 2022 [112]	realist-informed systematic review	absorption, adaptation, transformation; (includes reference to Blanchet et al. 2017 [88])

Most of the reviews refer to multiple levels or locations at which resilience can be addressed and assessed, and the importance of including stakeholders from service providers to governments to patients themselves in design and planning processes in order to build resilience. The Resilience in Healthcare group [113] in particular notes that patients are part of resilient responses, and draw from their analysis of multiple levels where ‘resilience characteristics’ can be found– ‘individual, team, management and organizational’. Other approaches take up the healthcare system components via the WHO, thus using a modular approach to the system as a whole (eg. Bayntun et al. 2012 [90]).

In terms of resilience aspects or phases, about half the reviews prior to 2021 draw out ‘preparedness’ as a notion covered by their included studies. Hess et al. 2012 [86] take up ‘adaptive capacity’, specifically in relation to climate change; and ‘adaptation’ is further covered by Zhong [91], Chamberland-Rowe et al. 2019 [95], Haldane et al. 2021 [79], while Turenne 2019 [25], Foroughi et al., [105, 107], Thu [109], Fridel et al. 2020 [98], Fleming [110] and Ismail [112], also make explicit mention of ‘transformation’ in addition to adaptation. Transformation and preparedness is also covered in the scoping review by Nuzzo et al. 2019 [94]. However, despite more recent reviews mentioning and identifying the concept of transformation in the studies they reviewed, they note that its application and use is less present in empirical work.

The key messages that stand out from these reviews is the lack of a common definition of resilience [25, 94, 98], and the scarce or underdeveloped use of learning and transformation as concepts operationalised in the empirical literature, which is also in line with our findings from the empirical literature. Furthermore, Nuzzo et al. 2019 point out the lack of implementation frameworks to translate resilience capacities into something that health system actors can employ in response to crises [94]. However, the more recent reviews have started to focus on the importance of operationalising resilience in practice (e.g. [2, 3]). Despite the turn toward community resilience (learning, empowerment) and socio-ecological resilience (self-organization, transformation) concepts being recognized as important—in contrast to the more linear, engineering resilience approaches—this theoretical turn has not (yet) resulted in the adoption of those concepts in practice.

## Discussion

**Fig. 9:**
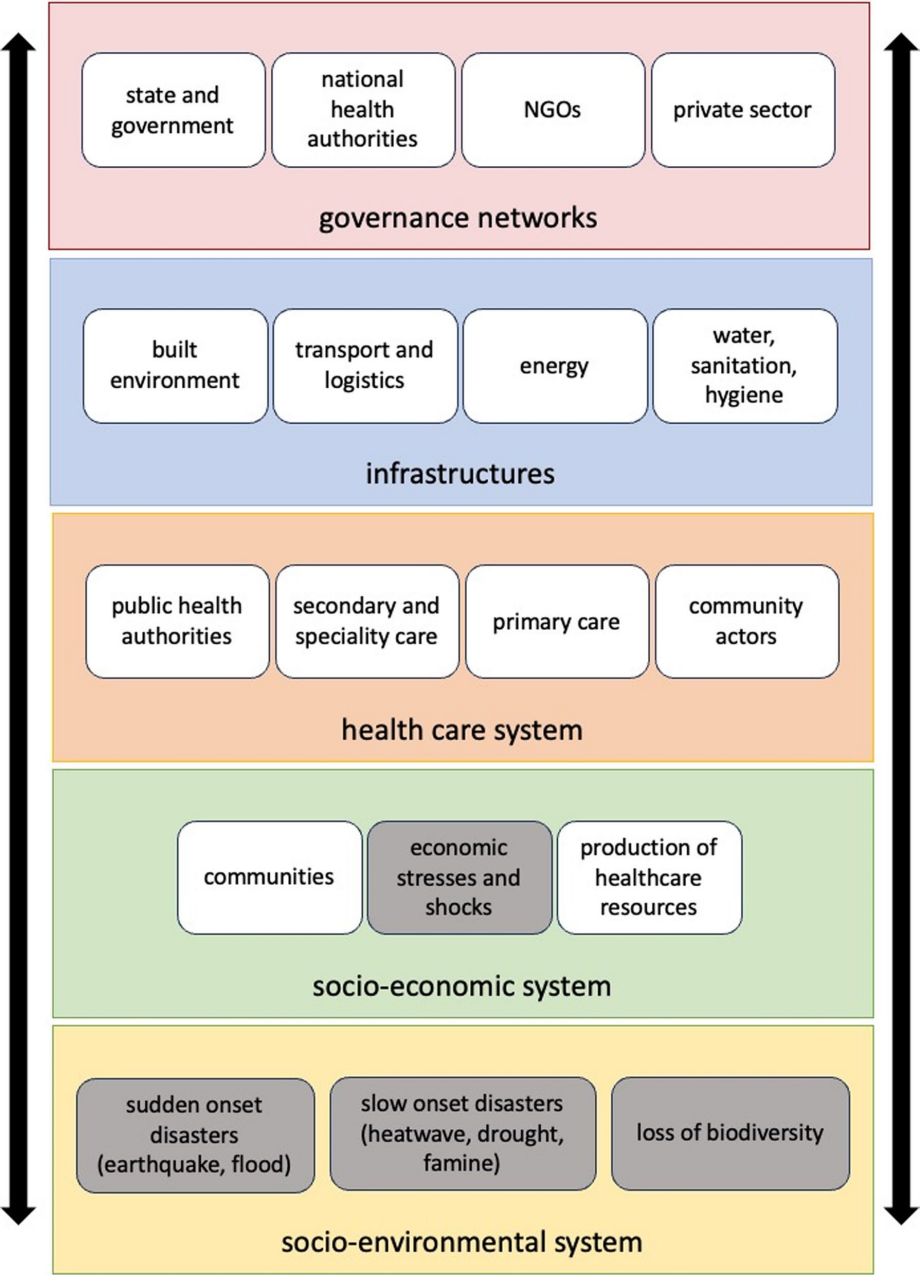
The health system as an adaptive complex system, and its interdependencies to other systems, grey boxes indicate shocks or stresses to health system

Health systems operate at the intersection of technical and social, and possibly social-environmental, systems. Figure 9 shows that they are complex systems, ranging from community services to highly specialised experts in hospitals and requiring local, regional, and national coordination. Moreover, they are embedded in the broader social-environmental, socio-economic, governance and infrastructural context (see Fig. 9). These systems shape and influence the shocks and stresses that the health system may be exposed to (black boxes in Fig. 9), but also determine its capacity to rapidly respond.

This breadth of applications and resilience challenges, as well as focal areas and time horizons considered is reflected in the health resilience literature: we found a wide variety of resilience approaches and schools, published in various health journals as well as in journals from neighbouring disciplines, most notably emergency management & computer science. Not surprisingly, applications that focus on physical infrastructure or built environment focus on resilience engineering, and bring approaches for improved planning, management, and operations of these infrastructures. For community, governance, or dealing with socio-ecological change, however, other concepts are vital that focus on self-organisation, learning, empowerment, and transformation. Because of the nature of the health system as a complex adaptive system of systems, the challenge to resilience in health is integrating these different facets and paradigms of resilience, because they all are vital to the health system.

In our study, we trace the roots of these resilience approaches to build and review the underlying schools of thought or paradigms and approaches to resilience. This produces a unique map, distinguished from previous reviews, placing concepts in alignment with the different subsystems of the health system to which they are applied. Further, by including both an analysis of empirical studies and review of reviews, we were able to validate our own preliminary findings: first, the empirical studies analyses served to identify what non-health domain concepts were present and in which patterns; and the reviews served to identify what resilience concepts were already being discussed in the literature. Putting the two together helped us formulate our conclusions and identify gaps in theoretical concepts from other domains present in the literature. Generally, our review shows that the field is fragmented, especially in terms of approaches and ‘schools of thought’ from non-health domains appealed to in the literature, as well as in terms of the health systems settings in which these are used. In the following paragraphs, we address the implications of these results.

First note that the intersection of health and resilience clearly has gained importance in the academic discourse with most papers published since 2018 (Fig. 4). This is a continued trend that confirms the findings of the review by Biddle et al. (2020) [1]. What our analysis further highlights is that most studies focus on either the resilience of health systems generally (and thereby responding to an external shock or stress), or on resilience within hospitals and thereby to the inherent uncertainty, volatility, and dynamics that are typical for the health system. Less attention has been given to community-based care, whether formal or informal, although the shift towards community did explicitly take place in reports on studies that focussed on responses to the Ebola outbreaks.

Second, conventionally, the resilience literature distinguishes three main capacities or outcomes: absorption, adaptation and transformation [1, 64]. However, our findings show that many of the empirical studies in health– especially those rooted in the risk, safety, and emergency management domain—focus on prevention and control as well as preparedness as key capacities and outcomes. While traditionally, these outcomes are considered as a part of risk management rather than resilience, the health resilience literature integrates both realms under the umbrella of resilience. The most used resilience aspect is ‘absorption’ or ‘rapidly bouncing back’ (Fig. 6), followed by preparedness and adaptation. What has previously received less attention (both in our empirical studies and in the reviews) is ‘transformation’, although this was pertinent in Ebola related studies. Outside of these studies, most focussed on preparedness and absorption, while it may be that COVID-19 has prompted a shift towards adaptation. However, the three combined aspects of absorbing, adapting and recovering seldom appear together, despite the resilience literature stressing the need to refer to all three.

Thirdly, of the various resilience paradigms influencing empirical work, ‘resilience engineering’ is the most prominent one mentioned, which is in line with the prominence of ‘absorption’ or ‘rapid response’ also highlighted. These concepts are closely linked as resilience engineering focuses on a single state of equilibrium or stability to which a resilient system rapidly returns after a shock. Despite being mentioned in most health subsystems in our included sample, it is especially prominent in hospital settings (Fig. 7). ‘Community resilience’ approaches find applications primarily in local and regional public health organisations, in studies of the health system as a whole (unspecified) and, not surprisingly, in formal and informal community actors. ‘Organisational resilience’ is applied to the different levels of public health organisations (local / regional) as well as secondary care (hospitals). The ‘social-ecological systems roots of resilience (relating to ‘transformation’ and multiple equilibria) find the least application, both in the health system as well as in hospitals.

Fourth, the different theoretical and conceptual roots also have implications for the approaches that are considered to build or improve resilience. While resilience engineering emphasises the need for coordination, robustness, flexibility, teams and different capacities, the literature that is rooted in community resilience stresses collaboration, trust, training, and leadership. Both resilience engineering and community resilience-oriented approaches acknowledge the need for information and surge capacity, as noted in the reviews we reviewed. Social capital-oriented literature is focusing primarily on the role of networks, while the organisational and social-ecological systems resilience literature stress the need for diversity.

As such, our findings show that the contemporary definitions of health systems resilience, along with the approaches to measure or build resilience, have not yet explicitly addressed important conceptual dilemmas or tensions apparent in the health resilience literature.

These conceptual dilemmas are related to:

- Paradigm & school: as health systems resilience is situated at the intersection of engineering, social, organisational, community, and socio-ecological systems resilience, bridges between the different schools of thought need to be found that allow for an integration of approaches and operationalisations. This concerns especially the question of a single equilibrium (restorative; bouncing back) versus multiple equilibria and transformational change.
- Temporality: intricately connected to the question of paradigm is the temporality considered. We find that the health resilience literature considers a wide variety of time horizons, even though there is a dominance of shorter time spans. The single equilibria approaches consider a relatively narrow frame to absorb and respond to a shock or stress. This is in contrast with the community and socio-ecological systems based approaches that stress the need to build trust, change, adapt and transform the health system. Without a clear definition of time horizons, measuring resilience becomes a conundrum, as different time horizons will lead to different results.
- Normativity: resilience is both used as a descriptive concept, to objectively measure how long it takes a system to recover performance (absorption), and as a normative concept, connected to terms such as inclusion, distributive justice, or sustainability. Especially the question of how values are or should be embedded into different resilience definitions and measurements remains open, making the underlying choices opaque and implicit. The lack of a clear discussion around the values that are conveyed through resilience, such as whose resilience is measured (and over what time horizon) has severe repercussions on our ability to measure resilience.
- Building resilience: connected to the lack of a clear stance on what constitutes resilience, or how it can be measured, is the broad variation of approaches that are introduced to improve resilience. While within engineering-oriented approaches, there is an emphasis on robustness, redundancy, and surge capacity, qualities such as trust, distributive justice, or adaptive capacity are stressed in the social-oriented resilience approaches. However, because the health system is an interconnected system-of-systems, what is needed is a toolkit of resilience building approaches that addresses different facets of resilience across different parts of the health system. Further, it is not known how the different approaches of building resilience would propagate and influence resilience in other areas of the health system.

These fundamental theoretical and conceptual dilemmas lead to a fragmentation of resilience concepts across the different realms of the health system (see Fig. 9) and make it difficult to develop a comprehensive definition of health systems resilience.

### Limitations

Our search was conducted for empirical articles up to June 2021, and for reviews up to June 2023. Given the ongoing COVID-19 pandemic and the continued interest in health system resilience, we are likely to be missing more recent articles that have covered the field further. Articles included in the descriptive statistics were tagged manually (for ‘threat’, ‘resilience concept’ and ‘part of the health system’) upon reading the title and abstract. We note that reading only titles and abstracts for half of our study is a potential limitation to our analysis. For example, it may be that the full text of these *n* = 87 articles would have resulted in different tags (for example, while the abstract may have referred to COVID-19 in its abstract, the full paper itself may have focussed on health system resilience more generally, without reference to a specific threat). Similarly, many articles were labelled as ‘no specific threat’, while in the full text itself, reference might have been made to specific threats such as Ebola or a natural disaster. To mitigate this type of error, where there was ambiguity in the abstract, the full text was read by one author so that the appropriate label was found for the article in question. Therefore, for the purposes of this portion of our results, where our aim was to understand the most salient and dominant theories, paradigms, approaches and threats relating to resilience, the need for full-text review was on a case-by-case basis and not required for the full set of articles. By contrast, for our second set of results, full-text review was conducted on the included reviews, since our intention was to give an overview of the discourses in the field addressed in existing reviews. Finally, we note that our databases were limited to those via Pubmed, and a wider search (e.g. including EMBASE) could have identified further studies to be included in our review, and to the English language, also limiting the global publications that could have contributed to our findings.

## Conclusions

While there are valuable lessons to learn about health system resilience through existing empirical work and reviews, the literature does not yet address important conceptual dilemmas relating to the underlying research paradigm or school, temporality, normativity, and building resilience. These fundamental theoretical and conceptual dilemmas and lead to a fragmentation of resilience concepts across the different realms of the health system make it difficult to develop a comprehensive definition or application of health systems resilience.

The health system is characterised by connections within and between the complex and adaptive sub-systems, ranging from community actors to local, regional, or national public health organisations to secondary care. Without a comprehensive definition and framework that captures these interdependencies, operationalising, measuring and improving resilience becomes challenging. We suggest that the different parts of the health systems should be conceptualized as networked subsystems. This will allow researcher to study resilience at the intersection of the different realms, and to understand how resilience propagates through different parts of the health system.

### Supplementary Information

**Additional file 1.** 

**Additional file 2.** 

**Additional file 3.** 

## Data Availability

All data included in the analysis in this study are available within the manuscript itself as supplementary files.

## Footnotes

1 https://www.unisdr.org/files/7817_7819isdrterminology11.pdf

2 https://www.ipcc.ch/sr15/chapter/glossary/
