# Supplementary material for: A resilience view on health system resilience: a scoping review of empirical studies and reviews
Source: BMC Health Serv Res. 2023 Nov 24;23:1297. doi: 10.1186/s12913-023-10022-8 (PMC10675888; doi:10.1186/s12913-023-10022-8)
Supplement: Supplementary file 1 — Additional file 1. [file 12913_2023_10022_MOESM1_ESM.pptx]

## Slide 1
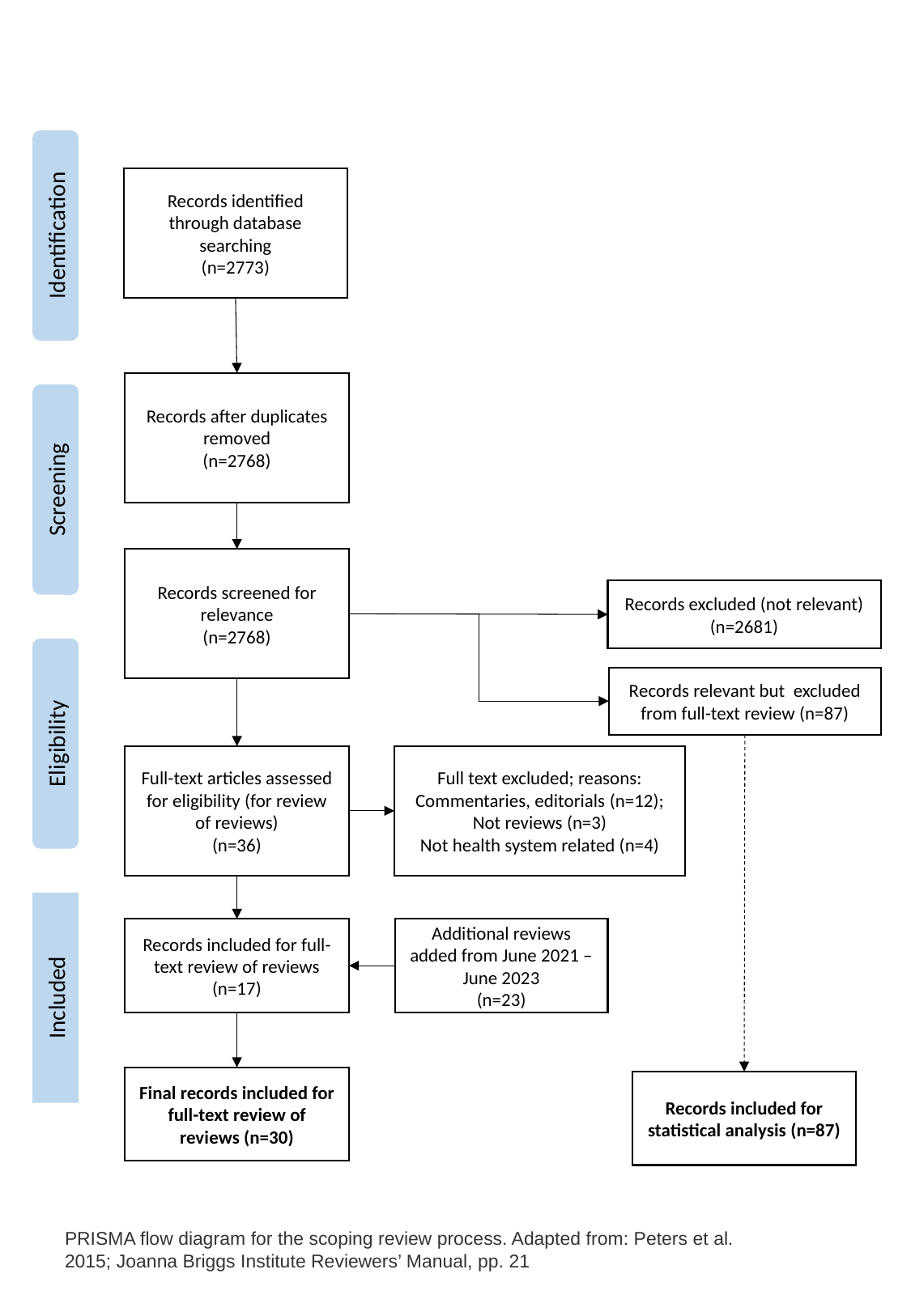

Records identified through database searching
(n=2773)
Identification
Records after duplicates removed
(n=2768)
Screening
Records screened for relevance
(n=2768)
Records excluded (not relevant)
(n=2681)
Records relevant but excluded from full-text review (n=87)
Eligibility
Full-text articles assessed for eligibility (for review of reviews)
(n=36)
Full text excluded; reasons:
Commentaries, editorials (n=12);
Not reviews (n=3)
Not health system related (n=4)
Records included for full-text review of reviews (n=17)
Additional reviews added from June 2021 – June 2023
(n=23)
Included
Final records included for full-text review of reviews (n=30)
Records included for statistical analysis (n=87)
PRISMA flow diagram for the scoping review process. Adapted from: Peters et al. 2015; Joanna Briggs Institute Reviewers’ Manual, pp. 21
